# Cutting-Edge Approaches in Arthroplasty: Before, during and after Surgery

**DOI:** 10.3390/jpm12101671

**Published:** 2022-10-08

**Authors:** Johannes Beckmann, David Barrett, Emmanuel Thienpont

**Affiliations:** 1 Clinic for Orthopaedic and Trauma Surgery, Hospital Barmherzige Brüder Munich, 80639 München, Germany; 2 Professor of Orthopaedic BioEngineering, School of Engineering Science, University of Southampton, Southampton SO17 1BJ, UK; 3 Saint-Luc University Clinics, Department of Orthopedics and Musculoskeletal Traumatology, Catholic University of Louvain (UCLouvain), Avenue Hippocrate 10, 1200 Brussels, Belgium

Personalised medicine was introduced in arthroplasty a long time ago with the aim of respecting each individual person for their unique personal characteristics in order to further improve outcomes. Compared to the early days of arthroplasty, the range of implant types, implant sizes, geometrical forms and implantation techniques has grown enormously in recent decades to deal better with the patients’ needs and their anatomy. Some of those technical evolutions were lauded as being the new holy grail, but most disappeared again or were assembled within the existing technique as a small upgrade.

The developments in hip arthroplasty seem to be less radical and more conservative, because of the longevity of the implants and the high satisfaction rates of patients. In knee arthroplasty, 20% to 30% remain dissatisfied, urging surgeons, designers and implant companies to find solutions to their problems.

In the past two decades, sizing issues, such as overhang and pain or downsizing and flexion instability, have been addressed. This led to the development of many different sizes with more representative anatomical aspect ratios and better surface matching in almost all modern implants, and culminated in true customised implants manufactured on a per-patient basis [1,2,3,4].

There has also been a renaissance in partial knee replacements, where resurfacing of only the diseased side of the knee can lead to better results. This could be performed in possibly up to 50% of patients instead of using totals. The counterpoint of a threefold higher revision rate of partials compared to total knees can be clearly disarmed by surgical experience and, lately, also for the first time by registry data. The German arthroplasty registry (EPRD) shows a non-inferiority of revision rates in those clinics performing a high volume of partial knees [5,6,7,8,9].

The latest debate concerning individuality in knee arthroplasty is the debate and trend towards personalized alignment. Each human being has their own unique type of coronal alignment. The idea is to approach this native alignment more closely with an oblique implant position. To be able to obtain these more complex goals in surgery, new technologies are needed, with the newest trend certainly being precision-enabling robots. Paradoxically, all these precision-enabling techniques such as robots, computer navigation or patient-specific instruments were used for a decade to avoid surgical outliers outside of the neutral mechanical axis. Now, they help to implant the same prostheses in different outlier positions. This new trend clearly shows that the target for coronal alignment has changed. It only remains to be proven that this improves the subjective outcome of the patient and will not lead to reduced survivorship. The new generation of robots combines the advantages and precision of navigation and robotics. It is indisputable that precision is higher with the help of these technologies compared to conventional jigs and eye-balling, even compared to experienced surgeons. However, thorough planning is mandatory to avoid a possible “trash in—trash-out” effect [10,11,12,13,14,15,16,17].

The difficulty with knee arthroplasty remains that all implants are made from metal and plastic and that they are supposed to substitute for cartilage, menisci and soft tissue structures such as ligaments. Furthermore, their shapes and radii of curvature were decided more than 40 years ago, mainly with the ambition of avoiding early failure of the materials. Today, these symmetrical implants ask for advanced technological tools to implant them asymmetrically into the native knee joint. Designing asymmetrical implants that respect the individual offsets of each part of the human knee might be a better evolution. Today, this remains difficult because of the logistics coming with this type of treatment and the high costs. However, if with the economy of scales and a higher volume of usage, the cost of goods can be reduced, this might be a more appealing concept for the future of arthroplasty. If this is combined with a robotic type of surgery, reducing the need for instrument sets and the patient-specific knee eliminates inventory, the value-chain of orthopaedics will have gone through its first new economic revolution in decades.

However, it is not only the “hardware” that makes the difference. The “software” of better peri-operative management of the patient has become a milestone in arthroplasty outcomes. Early mobilization, because of minimally invasive surgery, and improved pain and anaesthetic protocols are just some examples. Most of the dogmas that have existed in surgery for decades, and are transmitted from generation to generation of surgeons, were questioned and critically analysed. Postoperative drains were abandoned, the need for high pressure tourniquets was discussed and antifibrinolytic agents and local infiltration analgesia were introduced. The importance of clinical outcome for those changes are indisputable. While, a few decades ago, patients had to spend several weeks in hospital or even in bed following a joint replacement, arthroplasty has now become a procedure that is performed in outpatient surgery centres, where patients can leave the institution on the same day [18,19,20,21,22].

The growing importance of digitalization and collecting “big data” is relentless and one of the main topics for the future. The ultimate goal in arthroplasty will be to predict which technique and what system will help which patient with their unique anatomy. The collection of such “big data” physiologically and psychologically, pre-, intra- as well as postoperatively, together with expectations, satisfaction, capabilities and restrictions, will lead us to understand the real needs of our patients.

Although registers pool all arthroplasties performed, which initially does not seem to be very individual, national registers have to play a major role in documenting the quality of different implants and arthroplasty care overall, in order to describe best practice and report implant outliers. The registers have to be used for research and post-market surveillance, and register data may be a source for intelligent decision tools that can ultimately help to treat every individual patient better. This also helps in collecting “big data”. Predictive tools based on machine-learning algorithms could reform clinical practice, especially when combining machine-learning algorithms with data from nationwide arthroplasty registries [23,24,25,26,27,28,29,30,31,32].

Furthermore, early detection and prevention of arthritic changes in the joint, resulting in the need for arthroplasty, are also changing and will continue to do so. Radiological detection becomes more subtle with reduced radiation exposure and fast and broad availability by digitalization. The understanding of pathology and early treatment options improves almost day by day. Concerning arthroplasty, tissue engineering is just one aspect. Given the enormous increase in the risks of bone and cartilage defects with the increase in aging population, the current treatments available are insufficient for handling this burden, and the supply of donor organs for transplantation is limited. Therefore, tissue engineering is a promising approach for treating such defects. Advances in materials research and high-tech optimized fabrication of scaffolds have increased the efficiency of tissue engineering [33,34,35,36,37,38,39,40,41,42,43,44,45].

Pharmacological innovation might become important for the prevention of osteoarthritis in the near future, too. Surgeons remember how rheumatoid arthritis patients were their main segment of arthroplasty patients because of severe joint destruction and important deformities. Since the introduction of disease-modifying drugs, that segment of patients has severely changed [35,46,47,48]

One big issue and remaining problem for the coming years and decades to come is the revision arthroplasty of failed implants. Even with optimized implantation and improved materials, the more active patients operated on today will potentially need new surgeries in the future. The threshold age for arthroplasty is also coming down in patients operated on for sports traumatology in the past and who are experiencing early osteoarthritis. More surgeries in the elderly population and multi-operated patients will potentially lead to more peri-prosthetic infections, requiring revision surgeries. Issues such as instability and aseptic loosening often need to be addressed within the first years after the index procedure. The removal of implants, infection and osteolysis can lead to bone loss and the need for bone substitution with cones or resection-type implants. The number of implanted megaprostheses grows exponentially, as does the number of revisions. The socioeconomic burden is and will be immense [49,50,51,52,53,54].

This issue aims to address the cutting-edge topics concerning arthroplasty before, during and after surgery. It shows how surgeons are continuously looking for new ways to improve the outcomes for their patients and to share their knowledge with their community by sending these messages across as soon as possible so as to share innovation and improvements in care.

## References

[1] Beckmann J., Meier M.K., Benignus C., Hecker A., Thienpont E. (2021). Contemporary knee arthroplasty: One fits all or time for diversity?. Arch. Orthop. Trauma. Surg..

[2] Meier M., Janssen D., Koeck F.X., Thienpont E., Beckmann J., Best R. (2020). Variations in medial and lateral slope and medial proximal tibial angle. Knee Surgery, Sports Traumatol. Arthrosc..

[3] Meier M., Zingde S., Best R., Schroeder L., Beckmann J., Steinert A.F. (2019). High variability of proximal tibial asymmetry and slope: A CT data analysis of 15,807 osteoarthritic knees before TKA. Knee Surgery Sports Traumatol. Arthrosc..

[4] Meier M., Zingde S., Steinert A., Kurtz W., Koeck F., Beckmann J. (2019). What Is the Possible Impact of High Variability of Distal Femoral Geometry on TKA? A CT Data Analysis of 24,042 Knees. Clin. Orthop. Relat. Res..

[5] Berichte|EPRD. https://www.eprd.de/de/downloads-1/berichte.

[6] Beckmann J., Hirschmann M.T., Matziolis G., Holz J., Eisenhart-Rothe R.V., Becher C. (2020). Recommendations for unicondylar knee replacement in the course of time: A current inven-tory. Orthopade.

[7] Johal S., Nakano N., Baxter M., Hujazi I., Pandit H., Khanduja V. (2018). Unicompartmental Knee Arthroplasty: The Past, Current Controversies, and Future Perspectives. J. Knee Surg..

[8] Klasan A., Tay M.L., Frampton C., Young S.W. (2022). High usage of medial unicompartmental knee arthroplasty negatively influences total knee arthroplasty revision rate. Knee Surg. Sports Traumatol. Arthrosc..

[9] Hamilton T.W., Rizkalla J.M., Kontochristos L., Marks B.E., Mellon S., Dodd C.A., Pandit H.G., Murray D.W. (2017). The Interaction of Caseload and Usage in Determining Outcomes of Unicompartmental Knee Arthroplasty: A Meta-Analysis. J. Arthroplast..

[10] Thienpont E., Klasan A. (2021). The dissatisfied total knee arthroplasty patient. New technologies-the white knight in shining armor coming to their rescue?. Arch. Orthop. Trauma. Surg..

[11] Mathew K.K., Marchand K.B., Tarazi J.M. (2020). Computer-Assisted Navigation in Total Knee Arthroplasty. Surg. Technol. Int..

[12] Kayani B., Konan S., Ayuob A., Onochie E., Al-Jabri T., Haddad F.S. (2019). Robotic technology in total knee arthroplasty: A systematic review. EFORT Open Rev..

[13] Liu P., Lu F.-F., Liu G.-J., Mu X.-H., Sun Y.-Q., Zhang Q.-D., Wang W.-G., Guo W.-S. (2021). Robotic-assisted unicompartmental knee arthroplasty: A review. Arthroplasty.

[14] Subramanian P., Wainwright T., Bahadori S., Middleton R.G. (2019). A review of the evolution of robotic-assisted total hip arthroplasty. HIP Int..

[15] Jenny J.-Y., Picard F. (2017). Learning navigation—Learning with navigation. A review. SICOT-J.

[16] Siddiqi A., Horan T., Molloy R.M., Bloomfield M.R., Patel P.D., Piuzzi N.S. (2021). A clinical review of robotic navigation in total knee arthroplasty: Historical systems to modern design. EFORT Open Rev..

[17] Rivière C., Iranpour F., Auvinet E., Howell S., Vendittoli P.-A., Cobb J., Parratte S. (2017). Alignment options for total knee arthroplasty: A systematic review. Orthop. Traumatol. Surg. Res..

[18] Mariorenzi M., Levins J., Marcaccio S., Orfanos A., Cohen E. (2013). Outpatient Total Joint Arthroplasty: A Review of the Current Stance and Future Direction. Rhode Island Med. J..

[19] Pollock M., Somerville L., Firth A., Lanting B. (2016). Outpatient Total Hip Arthroplasty, Total Knee Arthroplasty, and Unicompartmental Knee Arthroplasty: A Systematic Review of the Literature. JBJS Rev..

[20] Jaibaji M., Volpin A., Haddad F.S., Konan S. (2020). Is Outpatient Arthroplasty Safe? A Systematic Review. J. Arthroplast..

[21] Vehmeijer S.B.W., Husted H., Kehlet H. (2017). Outpatient total hip and knee arthroplasty. Acta Orthop..

[22] Husted C., Gromov K., Hansen H.K., Troelsen A., Kristensen B.B., Husted H. (2019). Outpatient total hip or knee arthroplasty in ambulatory surgery center versus arthroplasty ward: A randomized controlled trial. Acta Orthop..

[23] Kremers H.M., Kremers W.K., Berry D.J., Lewallen D.G. (2015). Social and Behavioral Factors in Total Knee and Hip Arthroplasty. J. Arthroplast..

[24] Hafkamp F.J., de Vries J., Gosens T., Oudsten B.L.D. (2020). The Relationship Between Psychological Aspects and Trajectories of Symptoms in Total Knee Arthroplasty and Total Hip Arthroplasty. J. Arthroplast..

[25] Springer B.D., Sotile W.M. (2020). The Psychology of Total Joint Arthroplasty. J. Arthroplast..

[26] Siljander M.P., McQuivey K.S., Fahs A.M., Galasso L.A., Serdahely K.J., Karadsheh M.S. (2018). Current Trends in Patient-Reported Outcome Measures in Total Joint Arthroplasty: A Study of 4 Major Orthopaedic Journals. J. Arthroplast..

[27] Haeberle H., Helm J.M., Navarro S., Karnuta J.M., Schaffer J.L., Callaghan J.J., Mont M.A., Kamath A.F., Krebs V.E., Ramkumar P.N. (2019). Artificial Intelligence and Machine Learning in Lower Extremity Arthroplasty: A Review. J. Arthroplast..

[28] Gill S., Page R. (2019). From big data to big impact: Realizing the potential of clinical registries. ANZ J. Surg..

[29] Bedard N.A., Pugely A.J., McHugh M.A., Lux N.R., Bozic K.J., Callaghan J.J. (2018). Big Data and Total Hip Arthroplasty: How Do Large Databases Compare?. J. Arthroplast..

[30] El-Galaly A., Grazal C., Kappel A., Nielsen P.T., Jensen S.L., Forsberg J.A. (2020). Can Machine-learning Algorithms Predict Early Revision TKA in the Danish Knee Arthroplasty Registry?. Clin. Orthop. Relat. Res..

[31] Wang X., Hunter D.J., Vesentini G., Pozzobon D., Ferreira M.L. (2019). Technology-assisted rehabilitation following total knee or hip replacement for people with osteoarthritis: A systematic review and meta-analysis. BMC Musculoskelet. Disord..

[32] Varnum C., Pedersen A.B., Rolfson O., Rogmark C., Furnes O., Hallan G., Mäkelä K., de Steiger R., Porter M., Overgaard S. (2019). Impact of hip arthroplasty registers on orthopaedic practice and perspectives for the future. EFORT Open Rev..

[33] Qasim M., Chae D.S., Lee N.Y. (2019). Advancements and frontiers in nano-based 3D and 4D scaffolds for bone and cartilage tissue engineering. Int. J. Nanomed..

[34] Kwon H., Brown W.E., Lee C.A., Wang D., Paschos N., Hu J.C., Athanasiou K.A. (2019). Surgical and tissue engineering strategies for articular cartilage and meniscus repair. Nat. Rev. Rheumatol..

[35] Weber A.E., Bolia I.K., Trasolini N.A. (2020). Biological strategies for osteoarthritis: From early diagnosis to treatment. Int. Orthop..

[36] Kundu S., Ashinsky B.G., Bouhrara M., Dam E.B., Demehri S., Shifat-E-Rabbi M., Spencer R.G., Urish K.L., Rohde G.K. (2020). Enabling early detection of osteoarthritis from presymptomatic cartilage texture maps via transport-based learning. Proc. Natl. Acad. Sci. USA.

[37] Yu C., Zhao B., Li Y., Zang H., Li L. (2021). Vibrational Spectroscopy in Assessment of Early Osteoarthritis—A Narrative Review. Int. J. Mol. Sci..

[38] Onishi O., Ikoma K., Kido M., Kabuto Y., Ueshima K., Matsuda K.-I., Tanaka M., Kubo T. (2018). Early detection of osteoarthritis in rabbits using MRI with a double-contrast agent. BMC Musculoskelet. Disord..

[39] Zarringam D., Saris D., Bekkers J. (2019). The Value of SPECT/CT for Knee Osteoarthritis: A Systematic Review. CARTILAGE.

[40] Wyngaert T.V.D., Palli S.R., Imhoff R.J., Hirschmann M.T. (2018). Cost-Effectiveness of Bone SPECT/CT in Painful Total Knee Arthroplasty. J. Nucl. Med..

[41] Van der Bruggen W., Hirschmann M.T., Strobel K., Kampen W.U., Kuwert T., Gnanasegaran G., Van den Wyngaert T., Paycha F. (2018). SPECT/CT in Postoperative Painful Hip Arthroplasty. Semin. Nucl. Med..

[42] Weber M.-A., Merle C., Rehnitz C., Gotterbarm T. (2016). Modern Radiological Imaging of Osteoarthritis of The Hip Joint With Consideration of Predisposing Conditions. RöFo-Fortschritte auf dem Gebiet der Röntgenstrahlen und der bildgebenden Verfahren.

[43] Langer R., Vacanti J. (2015). Advances in tissue engineering. J. Pediatr. Surg..

[44] Bakhshandeh B., Zarintaj P., Oftadeh M.O., Keramati F., Fouladiha H., Sohrabi-Jahromi S., Ziraksaz Z. (2017). Tissue engineering; strategies, tissues, and biomaterials. Biotechnol. Genet. Eng. Rev..

[45] Koon K.T.V., Grenier D., Taborik F., Perrier A.-L., Mahieu-Williame L., Magnier L., Chuzel T., Contamin H., Chereul E., Beuf O. (2021). Comparison of high-resolution magnetic resonance imaging and micro-computed tomography arthrography for in-vivo assessment of cartilage in non-human primate models. Quant. Imaging Med. Surg..

[46] Sun Y., Zuo Z., Kuang Y. (2020). An Emerging Target in the Battle against Osteoarthritis: Macrophage Polarization. Int. J. Mol. Sci..

[47] Jones G.G., Clarke S., Harris S., Jaere M., Aldalmani T., de Klee P., Cobb J.P. (2019). A novel patient-specific instrument design can deliver robotic level accuracy in unicompartmental knee arthroplasty. Knee.

[48] Fusco G., Gambaro F.M., Di Matteo B., Kon E. (2021). Injections in the osteoarthritic knee: A review of current treatment options. EFORT Open Rev..

[49] Klug A., Gramlich Y., Rudert M., Drees P., Hoffmann R., Weißenberger M., Kutzner K.P. (2020). The projected volume of primary and revision total knee arthroplasty will place an immense burden on future health care systems over the next 30 years. Knee Surgery Sports Traumatol. Arthrosc..

[50] Klug A., Pfluger D.H., Gramlich Y., Hoffmann R., Drees P., Kutzner K.P. (2021). Future burden of primary and revision hip arthroplasty in Germany: A socio-economic challenge. Arch. Orthop. Trauma. Surg..

[51] Premkumar A., Kolin D.A., Farley K.X., Wilson J.M., McLawhorn A.S., Cross M.B., Sculco P.K. (2020). Projected Economic Burden of Periprosthetic Joint Infection of the Hip and Knee in the United States. J. Arthroplast..

[52] Rak D., Weißenberger M., Horas K., von Hertzberg-Bölch S., Rudert M. (2021). Mega-prostheses in revision knee arthroplasty. 2021, 50, 1011–1017. Der Orthopade.

[53] Melnic C.M., Lightsey H.M., Calderón S.A.L., Heng M. (2021). Megaprostheses in Nononcologic Hip and Knee Revision Arthroplasty. J. Am. Acad. Orthop. Surg..

[54] Tarazi J.M., Chen Z., Scuderi G.R., Mont M.A. (2021). The Epidemiology of Revision Total Knee Arthroplasty. J. Knee Surg..

